# Molecular characteristics and *Helicobacter pylori* infection rates in patients with gastric cancer in Western Poland: a comparative analysis of gastrectomy specimens across two decades

**DOI:** 10.3389/fonc.2026.1651941

**Published:** 2026-02-03

**Authors:** Jan Majewski, Marta Moszyńska, Kamila Stawczyk-Eder, Aldona Woźniak, Agnieszka Dobrowolska, Elżbieta Kaczmarek, Piotr Eder

**Affiliations:** 1 Department of Oncological Pathology, Poznan University of Medical Sciences, University Clinical Hospital, Poznan, Poland; 2 Department of Gastroenterology, Dietetics and Internal Medicine, Poznan University of Medical Sciences, University Clinical Hospital, Poznan, Poland; 3 Department of Clinical Pathomorphology, Poznan University od Medical Sciences, Poznan, Poland; 4 Department of Bioinformatics and Computational Biology, Poznan University of Medical Sciences, Poznan, Poland

**Keywords:** gastric cancer, gastrectomy, helicobacter pylori, histopathology, immunohistochemistry, proliferation index

## Abstract

**Introduction:**

Gastric cancer incidence and characteristics vary due to environmental factors, and technical advances facilitate early detection of the disease. This study aimed to assess whether significant socio-economic changes and technological advancements in Poland — one of the most rapidly developing countries worldwide — affected the molecular characteristics and detection rates of early gastric cancer sub-types in Western Poland.

**Methods:**

Ninety-two patients undergoing gastrectomy for gastric cancer in 1998–2002 and 2016–2020 were studied. Surgical specimens were re-analyzed for histopathological features, including tumor type, grade, and stage (up-dated World Health Organization [WHO] classification). Immunohistochemical markers (Ki-67, p53, E-cadherin, CD10, CD31, bcl-2) and antigens for Helicobacter pylori (H. pylori) and Epstein-Barr virus (EBV) were evaluated. Microsatellite instability (MSI) was assessed via PMS2/MSH2 protein expression.

**Results:**

The groups were comparable in age and gender, with male predominance. Histological features, H. pylori and EBV colonization, and most molecular markers showed no significant differences. However, Ki-67 proliferation index significantly increased in cancers diagnosed in 2016-2020, correlating with intestinal-type tumors and p53 expression. In this group, higher Ki-67 levels were also linked to H. pylori infection, microsatellite stability, and increased angiogenesis.

**Conclusions:**

Despite advancements in H. pylori research and technology over 20 years, no improvement was observed in H. pylori-positive tumor rates or early gastric cancer detection in Western Poland. Although molecular characteristics remained largely unchanged, the increased proliferation index in recently diagnosed cancers merits further study.

## 1 Introduction

Gastric cancer, despite the observed declining trends in incidence in many countries, remains a significant diagnostic and therapeutic challenge. In 2020, it was the fourth most common cause of cancer-related deaths worldwide (1). In Poland, this cancer is the fifth leading cause of cancer-related deaths in men and the eighth in women (2). This malignancy occurs almost twice as often in men as in women. The highest incidence rates in both sexes are observed in East Asia (including Japan and Mongolia), while in men, high rates are also reported in Western Asia (notably in Turkmenistan and Iran) (3). In the United States, the 5-year survival rate for gastric cancer is 31% (4).

Early diagnosis, being one of the most important prognostic factors impacting the overall survival, is hindered by the initially asymptomatic course of the disease and the nonspecific nature of symptoms in its early stages (5, 6). The most common symptoms generally include epigastric pain, a feeling of nausea, and fatigue, which, in most cases, tend to appear only when the cancer is already in an advanced stage (7). As a result, gastric cancer is still diagnosed frequently at a late stage of the disease, when palliative care becomes the only therapeutic option. In patients diagnosed earlier, combination of surgery and chemotherapy still belongs to the mainstay of treatment. In rare cases of disease limited to the superficial layers of the gastric wall without systemic involvement, endoscopic submucosal dissection (ESD) can serve as a single, curative procedure (6).

Although genetic predisposition is crucial in gastric cancer pathogenesis, the significant variation in the incidence of the disease across different countries and regions of the world indicates a crucial etiological influence of environmental factors. This was demonstrated, for example, in a study of Japanese populations who emigrated to Hawaii. The incidence of gastric cancer in the first generation of emigrants was lower than in those who remained in Japan. In each subsequent generation, the incidence continued to decline, although it remained higher than in the native Hawaiian population (8).

Modifiable environmental risk factors for gastric cancer include Helicobacter pylori (H. pylori) infection, low socioeconomic status, alcohol consumption, smoking, and an unhealthy diet (5). These factors particularly affect the intestinal type of gastric cancer according to the Lauren’s classification (9, 10). The specific mechanisms leading to the development of gastric cancer are not well understood. However, it appears that chronic inflammation resulting from the factors mentioned above may play a key role, leading to atrophic gastritis and intestinal metaplasia (11). This sequence, which may lead to dysplasia and, subsequently, the development of intestinal type of gastric cancer, is also known as the Correa’s cascade (10, 12). The pathogenesis of a diffuse type of gastric cancer is known to a significantly lesser extent.

Environmental factors, crucial in the pathogenesis of gastric cancer, change over the years and are subject to modification, which can significantly impact the incidence of gastric cancer and the biology and histology of the disease. However, there are still few studies exploring this issue. Considering the paucity of data in this topic, we decided to conduct a comparative analysis of the histological and biological characteristics of gastric cancers, including H. pylori infection status and Ki-67 proliferation index, across two distinct time periods in Western Poland. This region is part of a country which has experienced one of the most dynamic, on a global scale, radical environ-mental, political, civilizational, and economic changes over the past decades (13–15).

## 2 Materials and methods

### 2.1 Patients

This retrospective study included all consecutive patients with gastric carcinoma who underwent total gastrectomy with lymphadenectomy, in two time spans: 1998–2002 and 2016-2020, whose tissue specimens were examined in one Pathomorphological Reference University Centre in Poznan, Poland.

The exclusion criteria were as follows:

- any neoadjuvant treatment
- any distant metastasis diagnosed before or during surgery
- inability to classify the tumor according to the pathological Tumor-Node-Metastasis (pTNM) classification
- poor quality of tissue material
- any deviation from standardized processing of tissue specimens.

### 2.2 Histology and immunochemistry

All surgical specimens were processed in a standardized and routine manner, being fixed in 4% buffered formalin, then embedded in paraffin blocks and stored. For the purpose of the current study all stored specimens were sliced, stained with hematoxylin and eosin (HE) and assessed once again in detail.

The following histopathological features were assessed:

- histological type, according to the World Health Organization (WHO) 2019 classification (16). The specimens were classified according to clinical prognosis as classical type (this type included tubular adenocarcinoma, papillary adenocarcinoma, mucinous adenocarcinoma) and tumors with unfavorable prognosis (this type included adenocarcinoma with mixed subtypes, poorly cohesive carcinoma signet ring cell phenotype and other cell phenotype, undifferentiated carcinoma) (17)
- histological type according to the Lauren’s classification (18), including intestinal-type (type I) and non-intestinal-type (diffuse-type and mixed-type encompassing type II and III)
- histological grading according to the WHO 2019 classification (16) (low-grade G1/G2 vs high-grade G3 carcinomas) – this classification refers only to the tubular and papillary-type of gastric cancers
- staging according to the depth of invasion, including early type (involving mucosa and/or submucosa, irrespectively of the presence of regional lymph node metastases) and advanced type (involving at least muscularis propria)
- staging according to the pTNM classification from 2017 (19). For the purpose of the study gastric cancers were divided into two groups – according to the T feature (pT1a, pT1b, pT2 vs. pT3, pT4a, pT4b tumors) and according to the presence of regional lymph nodes metastases (pN0 vs. pN1, pN2, pN3a and pN3b).
- angioinvasion and neuroinvasion – assessed only in the case of at least submucosa infiltration (20).

Additionally, all specimens underwent immunohistochemical staining for the expression of following molecules: CD31, bcl-2, p53, Ki-67, CD10, E-cadherin, Epstein-Barr virus (EBV) and H. pylori antigen (**Supplementary Table S1**) (5, 21–28). **Table 1** specifies the methods used for the assessment and quantification of the expression of particular antigens. Moreover, the immunohistochemical expression of PMS2 and MSH2 proteins were assessed in order to detect microsatellite instability (**Table 2**) (29). Immunohistochemistry was performed according to standardized and validated protocols (**Supplementary Table S2**) (30).

**Table 1:** Tissue localization and methods used for the assessment of immunohistochemical (IHC) expression of molecules defining molecular characteristics of gastric cancers included in the analysis.

Parameter	Tissue localization	Method of quantification
Helicobacter pylori	Mucosa surrounding the tumor	Identification of spiral bacteria in IHC
Epstein-Barr Virus	Mucosa surrounding the tumor	Positive result: expression in ≥10% cells
p53	Tumor tissue	Mean % of positive cells assessed in three “hot spots”, magnification 400 x
Ki-67	Tumor tissue	Mean % of positive cells assessed in three “hot spots”, magnification 400 x
bcl-2	Tumor tissue	Positive result: expression in ≥10% cells
CD31	Tumor stroma	Mean number of CD31+ vessels assessed in three “hot spots”, magnification 400 x
CD10	Tumor tissue and stroma	Positive result: expression in ≥10% cells
E-cadherin	Tumor tissue	Positive result: expression in ≥10% cells

**Table 2:** Microsatellite instability status of gastric cancer tissues based on immunohistochemical expression of MSH2 and PMS2 proteins.

Microsatellite instability status	Gene inactivation	Protein	
		MSH2	PMS2
MSS	–	+	+
MSI	MSH2	X	+/x
MSI	PMS2	+/x	X

x: no expression of the protein in tumor tissue or expression seen in < 10% of cells. +: expression of the protein in tumor tissue seen in ≥ 10% of cells. MSI, microsatellite instability; MSS, microsatellite stability.

All histopathological assessments were performed independently by two qualified histopathologists with more than 10 years of experience in gastrointestinal pathology. In the case of quantitative assessments, the final results represented the mean assessed values. For qualitative and descriptive parameters in the case of any discrepancy in the interpretation of the results, team reassessment was conducted.

### 2.3 Statistical analysis

Statistical analysis was first performed through the Shapiro–Wilk test to verify the normality of data. Since the lack of the data normal distribution, the Mann-Whitney test was employed to compare the results between two time spans: 1998–2002 and 2016-2020. A chi-square distribution-based approach was used to compare two independent proportions (in %) of CD10 in tumor cells, CD10 in tumor stroma, E-cadherin and microsatellite instability (MSI) between 1998–2002 and 2016–2020 time periods. Associations between Ki-67 expressions and histological prognostic factors as well as the expressions of other biomarkers were tested by the chi-square test of independence. A correlation between Ki-67 and number of vessels in tumor stroma as well as between Ki-67 and p53 were verified by the Spearman rank correlation coefficient r. In Spear-man rank correlation a value of coefficient r greater than 0.7 is considered a strong correlation. Anything between 0.4 and 0.7 is a moderate correlation and anything less than 0.4 is considered a weak or no correlation. The statistical analysis was performed using the Statistica 13.3 PL software package (StatSoft, Poland). A value of P<0.05 was considered as statistically significant.

### 2.4 Bioethical considerations

Due to the retrospective character of this study, the requirement for informed consent was waived by the Bioethics Committee of the Poznan University of Medical Sciences (Decision No. 19/02/28).

## 3 Results

The final study group consisted of 92 patients who underwent surgical treatment due to gastric cancer in two 5-year time periods: 1998-2002 (n=47) and 2016-2020 (n=45). The subgroups were comparable in terms of age and gender (**Table 3**), with a significant predominance of male patients in both timespans (1998-2002: 31/47– 66% vs. 16/47 – 34%, p=0.03; 2016-2020: 33/45 – 73% vs. 12/45 – 27%, p=0.005).

**Table 3:** Differences in characteristics of treatment group and immunohistochemical test results between groups from 1998–2002 and 2016-2020.

Variable	Tumors diagnosed in 1998-2002	Tumors diagnosed in 2016-2020	p
Age, mean (SD)	65 (11) years	69 (11) years	0.11
Male, n (%)	31 (66%)	33 (73%)	0.7
Female, n (%)	16 (34%)	12 (27%)	0.46
H. pylori, n (%)	19/43 (44%)	15/40 (38%)	0.48
EBV, n (%)	12/43 (28%)	12/40 (30%)	0.7
Ki-67, median [IQR]	0.3 [0.18-0.42]	0.41 [0.15-0.8]	0.02
p53, median [IQR]	0.11 [0-0.8]	0.04 [0-0.8]	0.79
bcl-2, n (%)	12/43 (28%)	13/40 (32%)	0.84
Number of vessels in tumor stroma (CD31), mean (SD)	23 (20)	23 (16)	0.98
CD10 in tumor cells, n (%)	15/43 (35%)	18/40 (45%)	0.6
CD10 in tumor stroma, n (%)	19/43 (44%)	11/40 (28%)	0.16
E-cadherin, n (%)	43 (91%)	38 (84%)	0.33
MSI, n (%)	27 (57%)	24 (53%)	0.77

### 3.1 Comparison of histopathological characteristics

There were no significant differences in percentages of histological types according to the WHO 2019 classification between the study groups. Namely, 33/47 (70%) and 26/45 (58%) patients were diagnosed with classical type of gastric cancer in the timespan 1998–2022 and 2016-2020, respectively (p=0.4). At the same time, histological type of unfavorable prognosis was found in 30% (14/47 for the timespan 1998-2002) and 42% of cases (19/45 for the timespan 2016-2020). Detailed numbers of specific histological types in each timespan are shown in the **Supplementary Table S3**.

Comparison of other histological characteristics as well as disease staging (in which we also found no significant differences) is presented in **Table 4**.

**Table 4:** Comparison of histological characteristics and disease staging between gastric cancers diagnosed in 1998–2002 and 2016–2020.

Histological characteristics/disease staging	Tumors diagnosed in 1998-2002; n (%)	Tumors diagnosed in 2016-2020; n (%)	p
Intestinal type	33 (70)	26 (58)	0.4
Non-intestinal type	14 (30)	19 (42)	0.4
High-grade cancer	6 (20)	8 (35)	0.6
Low-grade cancer	24 (80)	15 (65)	0.14
Early cancer	4 (9)	5 (11)	sample too small to calculate
Advanced cancer	43 (91)	40 (89)	0.7
pT1/2	10 (21)	9 (20)	0.8
pT3/4	37 (79)	36 (80)	0.9
pN0	18 (38)	14 (31)	0.5
pN1/2/3	29 (62)	31 (69)	0.8
angioinvasion	22 (50)	22 (50)	-
neuroinvasion	17 (39)	13 (29)	0.4

### 3.2 Comparison of immunohistochemical characteristics

**Table 3** presents the differences in the immunohistochemical expression of chosen antigens between patients who underwent gastrectomy in the years 1998–2002 and 2016-2020.

We found significant differences in the expression of Ki-67 in gastric cancers from the timespan 1998–2002 when compared to 2016-2020. For this reason, we then com-pared associations between Ki-67 expression and histological prognostic factors as well as the expression of other biomarkers.

**Figure 1:**
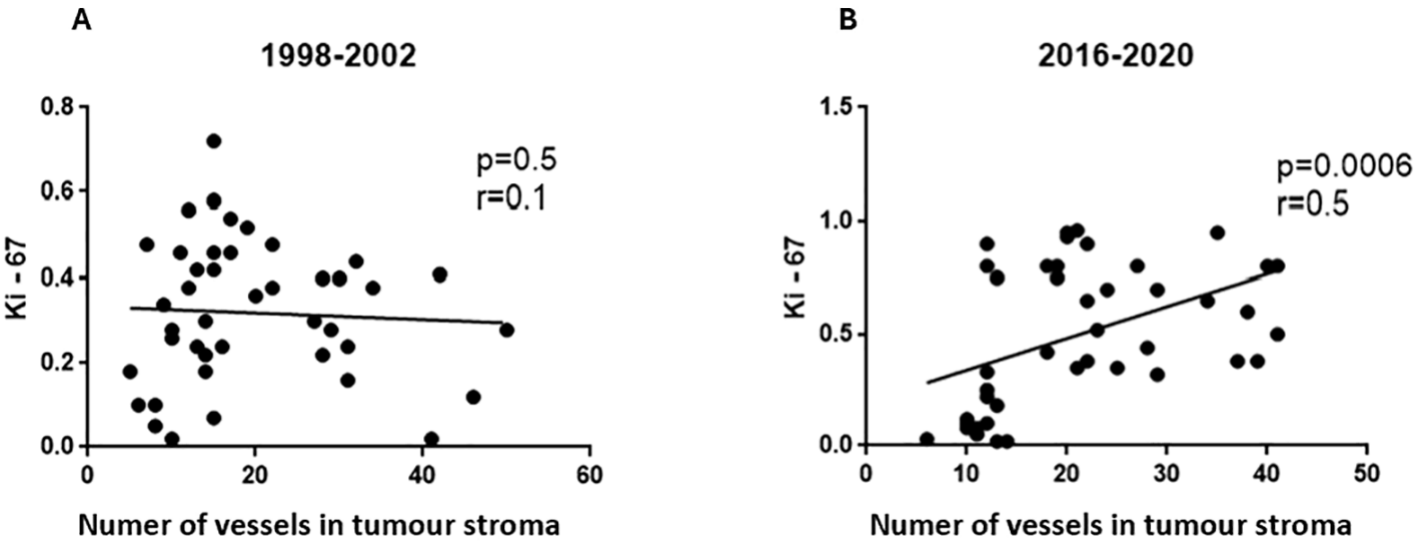
Correlations between CD31 expression in the endothelium of the stromal vessels of gastric carcinomas quantified as the number of CD31+ vessels in the large field of view and the proliferation index Ki-67. **(A)** 1998-2002 time span; **(B)** 2016-2020 time span.

**Figure 2:**
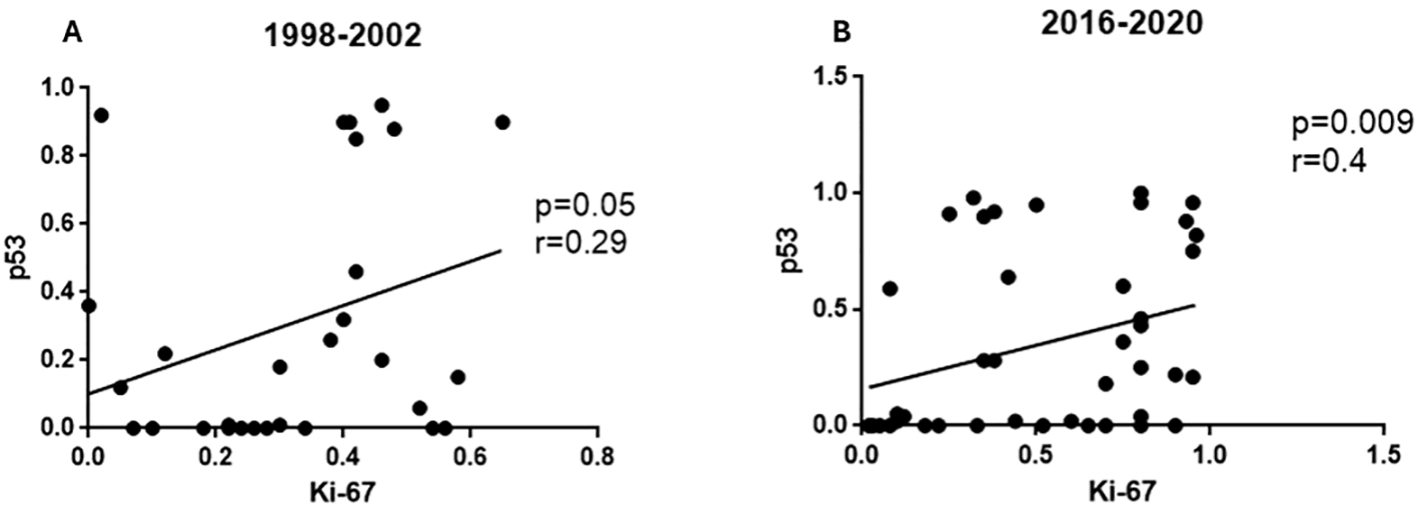
Correlations between immunohistochemical expression of p53 in gastric carcinoma cells and the Ki-67 proliferation index. **(A)** 1998-2002 time span; **(B)** 2016-2020 time span.

Ki-67 expression was significantly lower in non-intestinal types of gastric cancers in both timespans (**Table 5**). At the same time, Ki-67 expression was higher in H. pylori -infected patients but only in those operated in 2016-2020 (**Table 5**). Similarly, only in this subgroup of patients a significant correlation was found between Ki-67 and the number of proliferating vessels in the tumor stroma (**Figure 1**). In contrast to that, correlations between p53 expression and Ki-67 were found in the tumor cells in both study subgroups (**Figure 2**). Moreover, gastric cancers fulfilling the MSI criteria showed lower Ki-67 expressions in tissues coming from both timespans (**Table 5**).

**Table 5:** Differences in the proliferation activity of gastric cancer as assessed by Ki-67 expression between patients with different molecular and/or histologic features [data presented as means (SD)].

Molecular and/or histologic features	Ki-67 expression in gastric cancers diagnosed in 1998-2002	Ki-67 expression in gastric cancers diagnosed in 2016 – 2020
Intestinal-type vs. non-intestinal type according to the Lauren’s classification	0.35 (0.16) vs. 0.21 (0.16); p=0.03	0.59 (0.24) vs. 0.32 (0.39); p=0.04
Helicobacter pylori negative vs. Helicobacter pylori positive	0.28 (0.17) vs. 0.34 (0.19); p=0.35	0.39 (0.31) vs. 0.65 (0.26); p=0.007
Microsatellite instability vs. microsatellite stability	0.26 (0.19) vs. 0.37 (0.14); p=0.06	0.39 (0.31) vs. 0.62 (0.28); p=0.01

We did not find any associations in both study subgroups between the expression of Ki-67 and following characteristics: histological grading, gastric cancer staging (pTN), the presence of angio- and neuroinvasion, EBV status, CD10 expression (both in the tumor tissue and stroma), as well as E-cadherin and bcl-2 expression (data not shown).

## 4 Discussion

Our study showed that despite significant changes in many environmental factors in the last two decades, no differences were noted over two different time periods (1998–2002 vs 2016-2020) in terms of the histopathological characteristics of gastric cancers in the population studied. Considering the crucial prognostic role of early detection of gastric cancer, no increase in the percentages of early gastric cancer subtypes and no differences in tumor staging at the time of surgery as assessed by the pTNM classification among contemporarily diagnosed patients seem to be the most disappointing results from our study. These results are in contrast with data presented by Popiela et al. They compared two groups of operated patients from 1977–1999 and 1989-1999, showing a statistically significant increase in resected early-stage gastric cancers and a decrease in the number of patients operated with the most advanced disease (31). On the other hand, Borsh et al. analyzed 1161 patients operated on in Sweden due to gastric cancer from 1974-1991. Similarly to our study, they did not find a statistically significant differences in the detectability of early disease subtype throughout time (32). Conversely, Japan shows one of the highest diagnostic rates for early gastric cancer, reaching 50% compared to approximately 15% in Europe and 20% in the United States (10, 33, 34).

These differences in the detection rates of non-advanced gastric cancers can result, among others, from different strategies regarding screening programs or the quality of upper gastrointestinal endoscopy in different countries (35, 36). That is why, considering the data presented in our study and the fact that gastric cancer still belongs to the group of malignancies with the worst prognosis, it seems essential to promote strategies aimed at increasing the detection rates of early type of the disease. Common implementation of the recommendations given by the European Society of Gastrointestinal Endoscopy (ESGE), European Helicobacter and Microbiota Study Group (EHMSG), and European Society of Pathology (ESP) (37, 38) on endoscopic screening for gastric cancer among patients with severe atrophic gastritis and/or extensive intestinal metaplasia can be one of the proposed options. Another strategy would be to promote constant education in the quality of upper gastrointestinal endoscopy.

The lack of observed changes in the histological characteristics of the tumor (histological type, according to the WHO 2019 classification, Lauren’s classification, and histological grading) may be related to the influence of genetic and environmental factors. Genetic factors are not subject to modification, unlike the environment. Environmental factors are an essential part of the pathogenetic background of stomach cancer, as evidenced by noticeable differences in the incidence rates in various countries and regions worldwide (3). The last decades have represented two opposing trends regarding the environment and the lifestyle of populations in developed countries. On the one hand, there is a progression of air and water pollution, soil contamination, as well as the consumption of highly processed food rich in preservatives, and an obesity epidemic (10, 39). On the other hand, many countries have undertaken a series of pro-ecological initiatives, there has been a gradual improvement in people’s awareness of environmental protection (40), and there have been improvements in food storage conditions. There is a promotion of dietary habits that limit salt consumption and animal products and a dynamic popularization of Mediterranean or vegetarian diets (39, 41). The phenomena mentioned above also occur in the Polish population, where the last decades have been a period of exceptional, on a global scale, radical political, civilizational, and economic changes (42). Despite the relatively short period between the compared groups of patients in this study, they represent two different perspectives - the initial period of transformation (late 1990s) and the contemporary perspective, which seems sufficient to capture hypothetical trends regarding the differences in the characteristics of stomach cancers. It is worth noting that the issue of environmental influence is very complex and poorly understood. Analyzing the work of Yin and colleagues, who reviewed studies on the impact of environmental factors relevant to stomach cancer, one can conclude that environmental factors may have a slightly different relationship with the occurrence and prognosis of stomach cancer depending on the region under consideration (43). Moreover, these factors sometimes act synergistically and, in other cases, additively. It is, therefore, difficult to precisely identify which environmental elements are crucial. Nevertheless, the association with selected features characterizing the problem of stomach cancer is evident. According to the Lauren’s classification, there has also been a global decrease in the incidence of intestinal-type stomach cancers, which is associated, among other factors, with lifestyle, diet, and environmental factors (9, 10, 44). However, this trend was not observed in the current analysis.

In our study, immunohistochemical comparison analysis of the specimens be-tween the two predefined groups was also performed. This was feasible since the compared patient groups did not differ in general (age, gender) and histological characteristics. We found that Ki-67 proliferation index reached significantly higher values among patients operated on between 2016 and 2020 than among those under-going surgery between 1998 and 2002. Ki-67 is a protein engaged in all cell cycle phases except for the G0 phase, so a high proliferation index may indicate a more aggressive tumor growth (45). Regarding gastric cancer, we have evidence confirming this hypothesis. For example, Ko et al. evaluated the prognostic value of Ki-67 protein ex-pression in a group of 320 patients, finding that it has negative prognostic significance, especially for early and well-differentiated gastric cancers (46).

A statistically significant higher mean Ki-67 proliferation index was found among intestinal types of tumors according to the Lauren’s classification among patients from both timeframes. This observation may be somewhat surprising because, as mentioned above, a higher proliferation index, measured by Ki-67 expression, is associated with a worse prognosis, similar to the diagnosis of diffuse cancer compared to intestinal cancer. However, in accordance with this result, a statistically significantly higher mean Ki-67 proliferation index was seen in the group of patients with E-cadherin expression in tumor cells. This adhesive protein is closely associated with the intestinal type of gastric cancer, according to Lauren’s classification (47). Additionally, similar data was published by Tokuyasu et al. (48) in a more detailed analysis, Ko et al. also showed significantly higher Ki-67 expression in intestinal-type gastric cancers and hypothesized that Ki-67 might have different prognostic values in individual subtypes of gastric cancer being particularly useful in well-differentiated tumors (46). Therefore, the worse prognosis of poorly differentiated and diffuse-type cancers is determined by more complex and less obvious mechanisms, which are not reflected solely by Ki-67 expression, as is in the case of intestinal-type tumors.

Ki-67 was correlated with both p53 expression and MSI in the whole study group, nicely reflecting the current knowledge on their prognostic role in gastric cancer (49, 50). Moreover, a positive relationship was observed between the number of vessels in the tumor stroma and the Ki-67 index, but only in the group of patients operated on between 2016 and 2020. In the earlier surgical cohort, no trend approaching statistical significance was observed. Therefore, it can be assumed that the degree of proliferation of cancer cells in tumors diagnosed recently depends at least partly on tumor vascularity. Similar relationships are known for other types of gastrointestinal malignancies (51). However, the use of angiogenesis inhibitors and anti-proliferative agents in chemotherapy regimens for gastric cancer has not shown consistent efficacy, and their clinical relevance remains uncertain (52).

In this study, it was demonstrated that H. pylori -positive gastric cancers diagnosed only between 2016 and 2020 revealed a significantly higher tumor proliferation index Ki-67. Gucin et al. also found a statistically significant increase in the Ki-67 proliferation index in patients with gastric cancer infected with H. pylori (53). Similarly, Shiotani et al. showed that H. pylori infection increases the Ki-67 proliferation index and the apoptosis of gastric cells, which leads to intestinal metaplasia and thus to gastric cancer (54). This interesting relationship, which was not documented in our cohorts among cancers from the earlier period, may be related to the differences in H. pylori’s virulence, modified by environmental factors changing over time, which was well documented in experimental models (55).

In the above-mentioned context, the lack of a difference in the percentage of H. pylori-infected patients with gastric cancer between the two study periods may seem especially alarming considering that knowledge and recommendations regarding H. pylori eradication have significantly changed during this time. In 1997, worldwide guidelines for eradicating H. pylori as a key pathogenic factor in gastric cancer were introduced for the first time (56). These guidelines initially suggested treating infection only in selected cases, and awareness among physicians regarding the necessity and principles of therapy gradually and slowly increased. In contrast, 2016–2020 represents the period of the Maastricht V/Florence recommendations, according to which H. pylori eradication should be implemented in every diagnosed patient (57). Consequently, the percentage of H. pylori infections among the gastric cancers studied should de-crease, which was not the case. Therefore, it seems essential to undertake educational activities to further disseminate knowledge among healthcare workers regarding the adherence to currently applicable recommendations based on strong scientific evidence by international expert groups.

Currently, in many countries, including Poland, there is no population-based screening program for gastric cancer or H. pylori infection. There is ongoing debate and controversy regarding how such screening should be implemented—whether through endoscopic examinations or non-invasive H. pylori testing. Taking into ac-count the results and conclusions of our study, one could consider initiating screening with H. pylori testing, especially given its established role in gastric carcinogenesis. It should also be noted that endoscopy, although accurate, is expensive and technically demanding (58). Therefore, the inclusion of a gastric cancer screening strategy may be worth further consideration.

Our study has several limitations. Firstly, the study is retrospective in nature. Considering the significance of the problem and the results obtained, it would be interesting to conduct a long-term multicenter prospective study to verify the hypotheses presented in our analysis. Secondly, a limitation of our study is its single-center design. Although the research was conducted at the Pathomorphological Reference University Centre in Poznan — the largest facility of its kind in one of the most populous regions of Poland — the results may not be fully generalizable to the entire Polish population. It is also important to note that this study is primarily morphological and histopathological in nature, and was not designed to include clinical outcome correlations. Another limitation is the potential difference in patient selection between cohorts due to the increasing use of neoadjuvant therapy in more recent years. Although patients who received such treatment were excluded from the study, and tumor characteristics remained comparable between groups. Another limitations of our study is the selection of biomarkers. We focused on a specific panel including: CD31, Bcl-2, p53, Ki-67, CD10, E-cadherin, Epstein-Barr virus and H. pylori antigens, PMS2, and MSH2. We are aware, however, that there are additional valuable biomarkers, such as PD-L1 and HER2, which could have provided further insights and their evaluation represents an interesting direction for future research. In this study, MSI assessment was performed using PMS2 and MSH2 immunohistochemical evaluation. Although this approach provides a reliable overview of mismatch repair status, it does not include MLH1 and MSH6, which could offer additional complementary information. Other limitation is the lack of knowledge regarding a long-term follow-up of the patients included, which could allow us to assess the possible clinical implications of the study findings.

In conclusion, despite ongoing dynamic changes in environmental and socioeconomical status in Western Poland, we found no significant differences in histopathological characteristics of gastric cancers coming from two distant timeframes. On molecular level, however, cancer tissues obtained contemporarily seem to demonstrate more proliferative potential. From clinical point of view, no decrease in the percentages of non-advanced gastric cancers qualified for surgery and no improvement in the frequency of H. pylori-positive cases represent the most alarming findings. Although these observations should be further confirmed in larger, prospective cohorts, efforts to improve the quality of care with regard to early detection of gastric cancer and proper management of possible oncogenic factors should be implemented without delay.

## Data Availability

The raw data supporting the conclusions of this article will be made available by the authors, without undue reservation.

## References

[1] Sung H Ferlay J Siegel RL Laversanne M Soerjomataram I Jemal A . Global cancer statistics 2020: GLOBOCAN estimates of incidence and mortality worldwide for 36 cancers in 185 countries. CA A Cancer J Clin. (2021) 71:209–49. doi: 10.3322/caac.21660, PMID: 33538338

[2] Wojciechowska U Didkowska J . Nowotwory złośliwe w Polsce w 2021 roku. Cancers in Poland in 2021. Warsaw, Poland: Ministerstwo Zdrowia (2023). Available online at: https://onkologia.org.pl/sites/default/files/publications/2024-01/biuletyn_2021_1.pdf (Accessed January 29, 2025).

[3] Bray F Ferlay J Soerjomataram I Siegel RL Torre LA Jemal A . Global cancer statistics 2018: GLOBOCAN estimates of incidence and mortality worldwide for 36 cancers in 185 countries. CA A Cancer J Clin. (2018) 68:394–424. doi: 10.3322/caac.21492, PMID: 30207593

[4] Rawla P Barsouk A . Epidemiology of gastric cancer: global trends, risk factors and prevention. Prz Gastroenterol. (2019) 14:26–38. doi: 10.5114/pg.2018.80001, PMID: 30944675 PMC6444111

[5] Smyth EC Nilsson M Grabsch HI Van Grieken NC Lordick F . Gastric cancer. Lancet. (2020) 396:635–48. doi: 10.1016/S0140-6736(20)31288-5, PMID: 32861308

[6] Szczeklik A . Interna Szczeklika 2021. Kraków: Medycyna Praktyczna (2021). Polski Instytut Evidence Based Medicine.

[7] Mayo Clinic Staff . Stomach cancer. Available online at: https://www.mayoclinic.org/diseases-conditions/stomach-cancer/symptoms-causes/syc-20352438 (Accessed January 29, 2025).

[8] Kolonel LN Hankin JH Nomura AM . Multiethnic studies of diet, nutrition, and cancer in Hawaii. Princess Takamatsu Symp. (1985) 16:29–40. 3916200

[9] Crew KD Neugut AI . Epidemiology of gastric cancer. WJG. (2006) 12:354. doi: 10.3748/wjg.v12.i3.354, PMID: 16489633 PMC4066052

[10] Yoon H Kim N . Diagnosis and management of high risk group for gastric cancer. Gut Liver. (2015) 9:5–17. doi: 10.5009/gnl14118, PMID: 25547086 PMC4282848

[11] Dixon MF Genta RM Yardley JH Correa P . Classification and grading of gastritis: the updated sydney system. Am J Surg Pathol. (1996) 20:1161–81. doi: 10.1097/00000478-199610000-00001, PMID: 8827022

[12] Correa P Piazuelo MB . The gastric precancerous cascade. J Digest Dis. (2012) 13:2–9. doi: 10.1111/j.1751-2980.2011.00550.x, PMID: 22188910 PMC3404600

[13] Gomułka S . Poland’s economic and social transformation 1989–2014 and contemporary challenges. Cent Bank Rev. (2016) 16:19–23. doi: 10.1016/j.cbrev.2016.03.005

[14] Fredriksson E . Eastv Capital. Poland’s golden age of growth. Available online at: https://www.eastcapital.com/insights/Polands-golden-age-of-growth (Accessed January 29, 2025).

[15] Churski P . Economic geography series. In: Three Decades of Polish Socio-Economic Transformations: Geographical Perspectives, 1st. Springer International Publishing AG, Cham (2022).

[16] Nagtegaal ID Odze RD Klimstra D Paradis V Rugge M Schirmacher P . The 2019 WHO classification of tumours of the digestive system. Histopathology. (2020) 76:182–8. doi: 10.1111/his.13975, PMID: 31433515 PMC7003895

[17] Zhao X Li Y Yang Z Zhang H Wang H Lin J . Adenocarcinoma with mixed subtypes in the early and advanced gastric cancer. Caspa gokulan R, redaktor. Can J Gastroenterol Hepatol. (2021) 2021:1–13. doi: 10.1155/2021/8497305, PMID: 34746042 PMC8570884

[18] Laurén P . The two histological main types of gastric carcinoma: diffuse and so-called intestinal-type carcinoma: an attempt at a histo-clinical classification. Acta Pathol Microbiol Scandinavica. (1965) 64:31–49. doi: 10.1111/apm.1965.64.1.31, PMID: 14320675

[19] Brierley J Gospodarowicz MK Wittekind C . TNM classification of Malignant tumours. Eighth Vol. 253. . Chichester, West Sussex, UK Hoboken, NJ: John Wiley & Sons, Inc (2017).

[20] Nasierowska-Guttmejer A Majewski P Malinowska M . Stomach cancer. Morphology. Pol J Pathol. (2013) 64:s27–39. 24893506

[21] Ohtsuka J Oshima H Ezawa I Abe R Oshima M Ohki R . Functional loss of p53 cooperates with the *in vivo* microenvironment to promote Malignant progression of gastric cancers. Sci Rep. (2018) 8:2291. doi: 10.1038/s41598-018-20572-1, PMID: 29396430 PMC5797237

[22] Min KW Kim DH Son BK Kim DH Kim EK Seo J . A high ki67/BCL2 index could predict lower disease-free and overall survival in intestinal-type gastric cancer. Eur Surg Res. (2017) 58:158–68. doi: 10.1159/000448945, PMID: 28273657

[23] Almeida PR Ferreira VA Santos CC Rocha-Filho FD Feitosa RR Falcão EAA . E-cadherin immunoexpression patterns in the characterisation of gastric carcinoma histotypes. J Clin Pathol Lipiec. (2010) 63:635–9. doi: 10.1136/jcp.2010.076026, PMID: 20591914

[24] Huang WB . CD10-positive stromal cells in gastric carcinoma: correlation with invasion and metastasis. Japanese J Clin Oncol. (2005) 35:245–50. doi: 10.1093/jjco/hyi076, PMID: 15886270

[25] Ribatti D Guidolin D Marzullo A Nico B Annese T Benagiano V . Mast cells and angiogenesis in gastric carcinoma. Int J Exp Path. (2010) 91:350–6. doi: 10.1111/j.1365-2613.2010.00714.x, PMID: 20412338 PMC2962893

[26] Senchukova MA . Issues of origin, morphology and clinical significance of tumor microvessels in gastric cancer. WJG. (2021) 27:8262–82. doi: 10.3748/wjg.v27.i48.8262, PMID: 35068869 PMC8717017

[27] Bocian J Januszkiewicz-Lewandowska D . Zakażenia EBV – cykl życiowy, metody diagnostyki, chorobotwórczość. Postepy Hig Med Dosw. (2011) 65:286–98. doi: 10.5604/17322693.943104, PMID: 21677354

[28] Mathiak M Warneke VS Behrens HM Haag J Böger C Krüger S . Clinicopathologic characteristics of microsatellite instable gastric carcinomas revisited: urgent need for standardization. Appl Immunohistochem Mol Morphol. (2017) 25:12–24. doi: 10.1097/PAI.0000000000000264, PMID: 26371427 PMC5147042

[29] Pino MS Chung DC . Microsatellite instability in the management of colorectal cancer. Expert Rev Gastroenterol Hepatol. (2011) 5:385–99. doi: 10.1586/egh.11.25, PMID: 21651356

[30] Kumar GL Rudbeck L . Immunohistochemical staining methods. Carpinteria, California, USA: Dako North America, Incorporated (2009).

[31] Popiela T Kulig J Kolodziejczyk P Sierzega M . Changing patterns of gastric carcinoma over the past two decades in a single institution: clinicopathological findings in 1557 patients. Scandinavian J Gastroenterol. (2002) 37:561–7. doi: 10.1080/00365520252903116, PMID: 12059058

[32] Borch K Jönsson B Tarpila E Franzén T Berglund J Kullman E . Changing pattern of histological type, location, stage and outcome of surgical treatment of gastric carcinoma. Br J Surg. (2002) 87:618–26. doi: 10.1046/j.1365-2168.2000.01425.x, PMID: 10792320

[33] Noguchi Y Yoshikawa T Tsuburaya A Motohashi H Karpeh MS Brennan MF . Is gastric carcinoma different between Japan and the United States? Cancer. (2000) 89:2237–46. 11147594

[34] Kim GH Liang PS Bang SJ Hwang JH . Screening and surveillance for gastric cancer in the United States: Is it needed? Gastrointestinal Endoscopy. (2016) 84:18–28. doi: 10.1016/j.gie.2016.02.028, PMID: 26940296

[35] Hamashima C Shibuya D Yamazaki H Inoue K Fukao A Saito H . The Japanese guidelines for gastric cancer screening. Japanese J Clin Oncol. (2008) 38:259–67. doi: 10.1093/jjco/hyn017, PMID: 18344316

[36] Nam SY Choi IJ Park KW Kim CG Lee JY Kook MC . Effect of repeated endoscopic screening on the incidence and treatment of gastric cancer in health screenees. Eur J Gastroenterol Hepatol. (2009) 21:855–60. doi: 10.1097/MEG.0b013e328318ed42, PMID: 19369882

[37] Pimentel-Nunes P Dinis-Ribeiro M Ponchon T Repici A Vieth M De Ceglie A . Endoscopic submucosal dissection: European Society of Gastrointestinal Endoscopy (ESGE) Guideline. Endoscopy. (2015) 47:829–54. doi: 10.1055/s-0034-1392882, PMID: 26317585

[38] Dinis-Ribeiro M Areia M De Vries A Marcos-Pinto R Monteiro-Soares M O’Connor A . Management of precancerous conditions and lesions in the stomach (MAPS): guideline from the European Society of Gastrointestinal Endoscopy (ESGE), European Helicobacter Study Group (EHSG), European Society of Pathology (ESP), and the Sociedade Portuguesa de Endoscopia Digestiva (SPED). Endoscopy. (2012) 44:74–94. doi: 10.1055/s-0031-1291491, PMID: 22198778 PMC3367502

[39] Buckland G Travier N Huerta JM Bueno-de-Mesquita HB Siersema PD Skeie G . Healthy lifestyle index and risk of gastric adenocarcinoma in the EPIC cohort study: Healthy Lifestyle Index and Gastric Cancer Risk in EPIC. Int J Cancer. (2015) 137:598–606. doi: 10.1002/ijc.29411, PMID: 25557932

[40] Liu Z Anderson TD Cruz JM . Consumer environmental awareness and competition in two-stage supply chains. Eur J Operational Res. (2012) 218:602–13. doi: 10.1016/j.ejor.2011.11.027

[41] Sitarz R Skierucha M Mielko J Offerhaus J Maciejewski R Polkowski W . Gastric cancer: epidemiology, prevention, classification, and treatment. CMAR. (2018) 10:239–48. doi: 10.2147/CMAR.S149619 PMC5808709 29445300

[42] Namaki MSSE . Neo-globalization: premises, processes and the future. SIJBPG. (2017) 4:71. doi: 10.19085/journal.sijbpg040701

[43] Yin J Wu X Li S Li C Guo Z . Impact of environmental factors on gastric cancer: A review of the scientific evidence, human prevention and adaptation. J Environ Sci. (2020) 89:65–79. doi: 10.1016/j.jes.2019.09.025, PMID: 31892402

[44] Wu H Rusiecki JA Zhu K Potter J Devesa SS . Stomach carcinoma incidence patterns in the United States by histologic type and anatomic site. Cancer Epidemiol Biomarkers Prev. (2009) 18:1945–52. doi: 10.1158/1055-9965.EPI-09-0250, PMID: 19531677 PMC2786772

[45] Lazăr D Tăban S Sporea I Dema A Cornianu M Lazăr E . Ki-67 expression in gastric cancer. Results from a prospective study with long-term follow-up. Rom J Morphol Embryol. (2010) 51:655–61., PMID: 21103622

[46] Ko GH Go SI Lee WS Lee JH Jeong SH Lee YJ . Prognostic impact of Ki-67 in patients with gastric cancer—the importance of depth of invasion and histologic differentiation. Medicine. (2017) 96:e7181. doi: 10.1097/MD.0000000000007181, PMID: 28640099 PMC5484207

[47] Karayiannakis AJ Syrigos KN Chatzigianni E Papanikolaou S Karatzas G . E-cadherin expression as a differentiation marker in gastric cancer. Hepatogastroenterology. (1998) 45:2437–42., PMID: 9951940

[48] Tokuyasu N Shomori K Nishihara K Kawaguchi H Fujioka S Yamaga K . Minichromosome maintenance 2 (MCM2) immunoreactivity in stage III human gastric carcinoma: clinicopathological significance. Gastric Cancer. (2008) 11:37–46. doi: 10.1007/s10120-008-0451-1, PMID: 18373176

[49] Xiao LJ Zhao S Zhao EH Zheng X Gou WF Takano Y . Clinicopathological and prognostic significance of Ki-67, caspase-3 and p53 expression in gastric carcinomas. Oncol Lett. (2013) 6:1277–84. doi: 10.3892/ol.2013.1532, PMID: 24179508 PMC3813574

[50] Zhu L Li Z Wang Y Zhang C Liu Y Qu X . Microsatellite instability and survival in gastric cancer: A systematic review and meta-analysis. Mol Clin Oncol. (2015) 3:699–705. doi: 10.3892/mco.2015.506, PMID: 26137290 PMC4471517

[51] Ammendola M Sacco R Marech I Sammarco G Zuccalà V Luposella M . Microvascular density and endothelial area correlate with Ki-67 proliferative index in surgically-treated pancreatic ductal adenocarcinoma patients. Oncol Lett. (2015) 10:967–71. doi: 10.3892/ol.2015.3286, PMID: 26622606 PMC4509049

[52] Nakayama I Takahari D . The role of angiogenesis targeted therapies in metastatic advanced gastric cancer: A narrative review. JCM. (2023) 12:3226. doi: 10.3390/jcm12093226, PMID: 37176668 PMC10178968

[53] Gucin Z Çakmak T Bayyurt N Salih BA . Helicobacter pylori infection and relationship with gastric epithelial cell proliferation and apoptosis. Turkish J Med Sci. (2013) 43:739–46. doi: 10.3906/sag-1207-12

[54] Shiotani A Iishi H Ishiguro S Tatsuta M Nakae Y Merchant JL . Epithelial cell turnover in relation to ongoing damage of the gastric mucosa in patients with early gastric cancer: increase of cell proliferation in paramalignant lesions. J Gastroenterol. (2005) 40:337–44. doi: 10.1007/s00535-004-1549-9, PMID: 15870969

[55] Loh JT Beckett AC Scholz MB Cover TL . High-Salt Conditions Alter Transcription of Helicobacter pylori Genes Encoding Outer Membrane Proteins. Young VB, redaktor. Infect Immun. (2018) 86:e00626–17. doi: 10.1128/IAI.00626-17, PMID: 29229727 PMC5820969

[56] Current European concepts in the management of Helicobacter pylori infection . The maastricht consensus report. European helicobacter pylori study group. Gut. (1997) 41:8–13. doi: 10.1136/gut.41.1.8, PMID: 9274464 PMC1027220

[57] Malfertheiner P Megraud F O’Morain CA Gisbert JP Kuipers EJ Axon AT . Management of *Helicobacter pylori* infection—the Maastricht V/Florence Consensus Report. Gut. (2017) 66:6–30. doi: 10.1136/gutjnl-2016-312288, PMID: 27707777

[58] Januszewicz W Turkot MH Malfertheiner P Regula J . A global perspective on gastric cancer screening: which concepts are feasible, and when? Cancers. (2023) 15:664. doi: 10.3390/cancers15030664, PMID: 36765621 PMC9913879

