# Supplementary material for: Molecular characteristics and Helicobacter pylori infection rates in patients with gastric cancer in Western Poland: a comparative analysis of gastrectomy specimens across two decades
Source: Front Oncol. 2026 Feb 3;16:1651941. doi: 10.3389/fonc.2026.1651941 (PMC12909228; doi:10.3389/fonc.2026.1651941)
Supplement: Supplementary file 1 [file Table1.docx]

Supplementary Material

# Supplementary Tables

**Table S1.** Summary of immunohistochemical (IHC) markers used in the analysis – their function or role and significance in gastric cancer.

| Protein/ Immunohistochemical Marker | Function/Role | Significance in Gastric Cancer |
| --- | --- | --- |
| p53 | - regulates cell cycle - repairs DNA - activates apoptosis | - mutation or deletion within the gene encoding this protein is crucial in carcinogenesis - strong and diffuse expression of tp53 gene mutation marker is a negative prognostic factor |
| bcl-2 | - regulates apoptosis | - anti-apoptotic factor - has prognostic significance, especially with Ki-67 expression correlation |
| E-cadherin | - responsible for cell adhesion - present in epithelial cell membranes | - correlates with gastric cancer type according to Lauren’s classification - strong membranous reaction in intestinal type and loss of expression in diffuse type |
| CD10 | - proteolytic enzyme | - negative prognostic significance - correlates with depth of invasion and increased metastatic potential (especially in intestinal type) |
| CD31 | - adhesive protein in endothelial cells of blood vessels | - assesses average number of vessels in tumor stroma - increased angiogenesis correlates with worse prognosis and higher risk of distant metastases |
| Epstein-Barr virus | - influences the risk of developing gastric cancer | - significant risk factor, especially for intestinal type |
| Helicobacter pylori | - influences the risk of developing gastric cancer | - significant risk factor, especially for intestinal type - crucial subject to diagnosis and eradication |
| Microsatellite instability | - results from faulty repairing mismatched bases formed during DNA replication - disturbances within one or more genes: MSH2, PMS2, MLH1, MSH6 | - significant prognostic factor - gastric cancers with MSI have better prognosis than stable cancers |

**Table S2.** Summary of protocol of immunohistochemical (IHC) study procedures and marker evaluations.

|  | Details |
| --- | --- |
| Proteins/Markers Studied | p53, bcl-2, Ki-67, CD10, CD31, E-cadherin, PMS2, MSH2, Helicobacter pylori antigen, Epstein-Barr virus antigen |
| Tissue Preparation | - Fixed in 4% buffered formalin - Embedded in paraffin - Cut into 4µm sections - Mounted on SuperFrost®Plus slides (Menzel Gläser®) |
| Deparaffinization, Rehydration & Epitope Retrieval | - Performed in PT LINK water bath (Dako®) at 97°C for 20 min - *Low pH EnvisionFlex Target Retrieval Solution* (Dako®) for Ki-67 - *High pH EnvisionFlex Target Retrieval Solution* (Dako®) for other proteins |
| Staining Process | - Incubated in Wash Buffer (Dako®) for 10 min - Machine staining with Autostainer Link 48 - Dako® - Using visualisation kit - EnVisionFlex Mini Kit, High pH (Link) (Dako®) |
| Antibodies Used | - Monoclonal mouse antibodies (Agilent/Perlan/Dako®): anti-p53 (clone DO-7); bcl-2 (clone 124); Ki-67 (MIB1); anti-CD10 (clone 56C6); anti-CD31 (clone JC70A); anti-E-Cadherin (clone NCH38); anti-PMS2 (clone EP51); anti-MSH2 (clone FE11); anti-EBV, LMP (clone CS.1-4); - Polyclonal antibody: anti-*Helicobacter pylori;* - All antibodies were RTU (ready to use) |
| Antibody Enhancement | - Mouse linker used for: anti-p53, anti-CD10, anti-CD31, anti-E-Cadherin, anti-MSH2 |
| Incubation with Antibodies | 20 minutes |
| Visualization | 3,3`-diaminobenzidine (DAB) |
| Counterstaining | - Mayer's hematoxylin - Dehydration - Cover-slipped with DPX. |
| Controls | Dedicated positive control for each antibody, performed separately for both time groups. |
| Analysis | Conducted by two pathologists, discrepancies resolved by joint review. |
| Evaluation of Markers | Each marker was evaluated simultaneously for both time groups. |

**Table S3.** Histological types of gastric cancer diagnosed in the two study periods, classified ac-cording to WHO (2019).

| **Histological type (WHO classification)** | **1998–2002;**  **number of cases (n)** | **2016–2020;**  **number of cases (n)** |
| --- | --- | --- |
| Tubular adenocarcinoma | 27 | 22 |
| Papillary adenocarcinoma | 0 | 1 |
| Mucinous adenocarcinoma | 3 | 2 |
| Poorly cohesive carcinoma (signet-ring cell type) | 6 | 6 |
| Poorly cohesive carcinoma (other cell type) | 1 | 4 |
| Mixed adenocarcinoma | 5 | 4 |
| Undifferentiated carcinoma | 2 | 5 |
| Tubular adenocarcinoma (intramucosal carcinoma) | 3 | 1 |
